# Supplementary material for: Label-Free DNA Biosensor Using Modified Reduced Graphene Oxide Platform as a DNA Methylation Assay
Source: Materials (Basel). 2020 Nov 3;13(21):4936. doi: 10.3390/ma13214936 (PMC7663213; doi:10.3390/ma13214936)
Supplement: Supplementary file 1 [file materials-13-04936-s001.pdf]

*Supplementary*

# Label-Free DNA Biosensor Using Modified Reduced Graphene Oxide Platform as a DNA Methylation Assay

## 1. Fourier transform infrared (FTIR) characterization

FTIR spectra were performed by an FTIR Spectrometer (INVENIO R), equipped with a platinum attenuated total reflectance (ATR) module with a diamond ATR accessory A225/Q (Bruker Optic Inc., Billerica, MA, USA). Fixed load was applied to the small amount of sample to ensure full contact of solid with the diamond ATR. Solid samples were analyzed in lyophilized form. Before each measurement, background spectra were collected. Spectra were recorded at 25 °C from 4000 to 400 $cm^{-1}$ at a resolution of 2 $cm^{-1}$. Each spectrum was acquired by merging 128 interferograms. For the infrared (IR) spectra, we used a Bruker OPUS 8.1 (Bruker Optic Inc., Billerica, MA, USA) g and the JDXview v0.2 software was applied for spectra evaluation.

## 2. FTIR study of GO decorated with NPs

The efficiency of GO modifications with NPs loading was investigated by FTIR. The FTIR analysis (Figure 5) showed the presence of O–H as a broad vibration peak between (3300 $cm^{-1}$). A sharp peak of C=C (1630 $cm^{-1}$), weak band of COOH at (1200 $cm^{-1}$), and weak deformation of the C–O–C bond at 1050 $cm^{-1}$) present on the GO. The broad peak of O–H present in GO confirmed a high degree of oxidation [1–3]. FTIR analysis detected changes in the functional groups of synthesized rGO and derivatives of rGO-metal composites, which correspond to the elimination of functional groups due to reduction. In rGO, there was reduction in the vibration bending of O–H at (3300 $cm^{-1}$). Moreover, there was a sharp peak increase in the C=C at 1630 $cm^{-1}$) when compared to GO. In addition, the deformed peak was due to C–O–C at 1050 $cm^{-1}$. The synthesized different rGO-metal composites possessed all the described vibration and deformation bending at the wavelength similar to the rGO. This result clearly demonstrates the presence and reduction of GO into rGO which were further modified to form rGO-metal composites

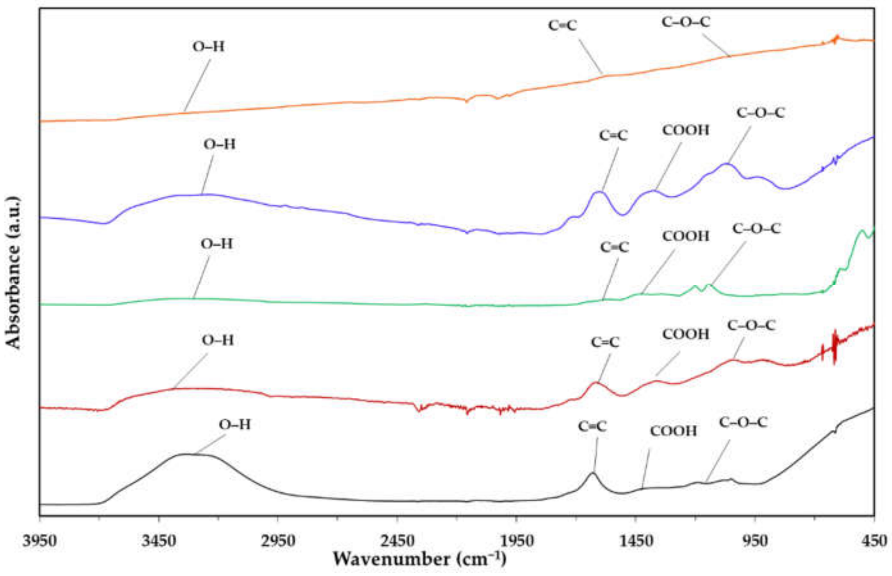


**Figure S1.** Fourier transform infrared (FTIR) spectra of GO (black) and prepared nanocomposites: rGO (red), rGO-CuNPs (green), rGO-AuNPs (blue) and rGO-AgNPs (orange).

## References


1. Zhang, X.; Guo, Q.; Cui, D.-X. Recent Advances in Nanotechnology Applied to Biosensors. *Sensors* **2009**, *9*, 1033–1053, doi:10.3390/s90201033.
2. Bao, J.; Geng, X.; Hou, C.; Zhao, Y.; Huo, D.; Wang, Y.; Wang, Z.; Zeng, Y.; Yang, M.; Fa, H.-B. A simple and universal electrochemical assay for sensitive detection of DNA methylation, methyltransferase activity and screening of inhibitors. *J. Electroanal. Chem.* **2018**, *814*, 144–152, doi:10.1016/j.jelechem.2018.02.060.
3. Jamróz, E.; Kopel, P.; Tkaczewska, J.; Dordević, D.; Jančíková, S.; Kulawik, P.; Milosavljevic, V.; Dolezelikova, K.; Smerkova, K.; Svec, P.; et al. Nanocomposite Furcellaran Films—the Influence of Nanofillers on Functional Properties of Furcellaran Films and Effect on Linseed Oil Preservation. *Polymers* **2019**, *11*, 2046, doi:10.3390/polym11122046.
